# Antigen presentation at the brain barriers in multiple sclerosis

**DOI:** 10.4103/NRR.NRR-D-25-00206

**Published:** 2025-07-05

**Authors:** Joshua Brands, Jeroen Bogie, Bieke Broux

**Affiliations:** University MS Center, Campus Diepenbeek, Diepenbeek, Belgium; Neuro-Immune Connections and Repair Lab, Department of Immunology and Infection, Biomedical Research Institute, Hasselt University, Diepenbeek, Belgium

Loss of immune tolerance to central nervous system (CNS) antigens lies at the heart of multiple sclerosis (MS), the most common chronic autoimmune disease of the CNS. MS affects nearly 2 million people worldwide and is characterized by focal areas of demyelination, inflammation, axonal injury, and neurodegeneration (Bronge et al., 2022; Magliozzi et al., 2023). The condition leads to symptoms such as numbness, muscle weakness, fatigue, visual impairment, and cognitive decline (Magliozzi et al., 2023). Although autoreactive T cells are considered to be primed in peripheral lymphoid tissues initially, it is the local reactivation of these T cells upon encountering CNS antigens that critically drives the inflammatory response within the CNS (Koch et al., 2022). It has become increasingly apparent that brain barriers, such as the blood–brain barrier (BBB), the blood–cerebrospinal fluid barrier (BCSFB), and the meninges, are central to modulating the immune response and driving MS pathology, potentially by facilitating antigen presentation and thus T cell reactivation (Ampie and McGavern, 2022; Magliozzi et al., 2023; Zierfuss et al., 2024). In this perspective, we examine current evidence on how antigen presentation occurs at major brain barrier sites and their contribution to triggering MS onset and progression.

**Antigen triggers in multiple sclerosis:** The leading hypothesis states that autoreactive T cells are initially primed in the periphery. Although the exact mechanism remains unclear, this priming may occur through infectious agents via molecular mimicry or CNS antigen presentation in the cranial or cervical lymph nodes (Ampie and McGavern, 2022). Once primed, myelin-reactive T cells accumulate at brain barrier sites, where they are reactivated by local dendritic cells as well as by other antigen-presenting cells (APC), including microglia, infiltrating macrophages and B-cells (Kivisakk et al., 2009; Ampie and McGavern, 2022; Jain and Yong, 2022; Magliozzi et al., 2023; Xu et al., 2024). While the exact disease-inducing peptides in MS have yet to be conclusively defined, several myelin-derived proteins consistently emerge as candidate auto-antigens (Bronge et al., 2022). Notably, myelin basic protein, proteolipid protein, myelin-associated glycoprotein, and myelin oligodendrocyte glycoprotein, all integral components of the myelin sheath are frequently observed in studies of MS patients, implying a potential role in the pathology (Bronge et al., 2022). Beyond these myelin-derived peptides, non-myelin antigens have been suggested as disease-inducing entities in MS. For instance, a recent study identified four novel antigens: fatty acid-binding protein 7, prokineticin-2, reticulon-3, and synaptosomal-associated protein 91, which all showed HLA-DR restriction (Bronge et al., 2022). Finally, viral antigens, mainly those from the Epstein-Barr virus (EBV), have been linked to MS pathogenesis (Lanz et al., 2022; Magliozzi et al., 2023). EBV is a human herpesvirus and is proposed to trigger MS through molecular mimicry (Lanz et al., 2022; Magliozzi et al., 2023). Specifically, EBV nuclear antigen 1 was recently shown to share structural similarities with the CNS protein GlialCAM, and both antigens were found to be recognized by clonally expanded B cells in the CNS (Lanz et al., 2022; Magliozzi et al., 2023). The compelling link between EBV infection and MS pathogenesis is supported by strong epidemiological and biological evidence indicating that EBV exposure significantly increases the risk of developing MS. Nonetheless, given the prevalence of EBV infection in adults (reaching 99%), it should not be considered as the sole disease-initiating factor.

These studies highlight the potential of multiple antigens to trigger MS and suggest a potential role for molecular mimicry in the activation of autoreactive lymphocytes. Notably, these autoreactive T cells can also be detected in healthy individuals, highlighting an intact peripheral tolerance system, suggested to be mainly mediated by regulatory T cells. However, in MS patients, this safeguard appears to be compromised, allowing for uncontrolled reactivation of autoreactive T cells that contribute to the disease pathology.

**Figure 1:**
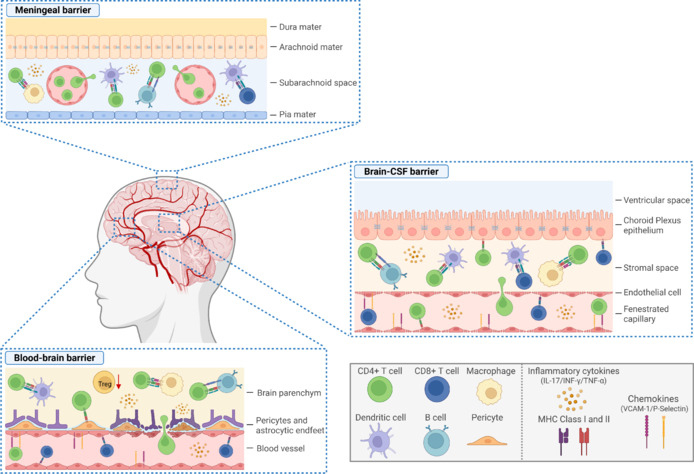
Antigen presentation at the barriers of the central nervous system. Created with BioRender.com. CSF: Cerebrospinal fluid; IL: interleukin; INF: interferon; MHC: major histocompatibility complex; TNF: tumor necrosis factor; VCAM-1: vascular cell adhesion molecule 1.

**Brain barriers as sites of antigen presentation:** Recent insights now underscore that the brain barriers are not mere physical separators but also dynamic immunological interfaces, facilitating antigen presentation and immune cell infiltration in MS (**Figure 1**).

First, the BCSFB, formed by the choroid plexus (CP) epithelium, is suggested to be the initial gateway for immune cell entry, most notably for Th17 cells, which are critical drivers for early MS pathology (Xu et al., 2024). Unlike the other brain barriers, the CP possesses fenestrated endothelial cells that allow for greater interaction between the peripheral circulation and the CNS (Ampie and McGavern, 2022). Importantly, recent studies indicate that CP epithelial cells are not merely passive structural components, they actively facilitate the immune response during neuroinflammation (Xu et al., 2024). Upon an inflammatory stimulus, these epithelial cells upregulate adhesion molecules and secrete a variety of chemokines that recruit neutrophils and monocytes (Xu et al., 2024). Furthermore, within the CP, different types of APC, including dendritic cells, macrophages, B cells, and other non-professional APCs, efficiently process CNS antigens (Xu et al., 2024). This early antigen presentation and the presence of a permeable barrier are essential for the priming, activation, and migration of Th17 cells, thereby setting the stage for the broader autoimmune response (Xu et al., 2024). Moreover, as inflammation advances and neural tissues are damaged, additional self-antigens are released and can be presented at the BCSFB and other barriers and potentially leak into the periphery, promoting epitope spreading (Bronge et al., 2022). This broadening of the antigenic repertoire may be a critical early event in MS pathogenesis, initiating disease processes years before clinical onset (Bronge et al., 2022).

Second, the BBB is the most extensively studied barrier between the brain parenchyma and the bloodstream. Traditionally recognized for its restrictive nature, it is profoundly altered in inflammatory conditions (Ampie and McGavern, 2022; Zierfuss et al., 2024). Formed by tightly joined endothelial cells and supported by pericytes and astrocytic endfeet, the BBB normally limits immune cell entry and maintains CNS homeostasis (Ampie and McGavern, 2022; Zierfuss et al., 2024). However, in MS, inflammation induces upregulation of adhesion molecules and disrupts tight junctions, leading to barrier breakdown (Ampie and McGavern, 2022; Zierfuss et al., 2024). It is hypothesized that this happens after the initial influx of Th17 cells via the CP, through local secretion of cytokines such as interleukin (IL)-17 and IL-22, which induce BBB breakdown. This disruption not only permits a greater influx of peripheral immune cells but also facilitates the exit of CNS antigens into the circulation (Aydin et al., 2023; Zierfuss et al., 2024). In this setting, various APCs, including dendritic cells, B cells, macrophages, pericytes, and activated endothelial cells further amplify antigen presentation (Aydin et al., 2023). New findings suggest that these activated endothelial cells, beyond their structural role in maintaining the BBB, also contribute to antigen presentation in MS (Aydin et al., 2023). They appear to be capable of processing and cross-presenting CNS antigens to CD8^+^ T cells via major histocompatibility complex (MHC) class I on the luminal side, facilitating their migration across the BBB and further contributing to its breakdown (Aydin et al., 2023). Similarly, pericytes have been implicated in MS pathology, exhibiting increased expression of adhesion molecules and MHC class II, which enables them to interact with and activate CD4⁺ T cells, thereby promoting immune cell infiltration (Koch et al., 2022). This highlights that, in addition to professional APCs, local barrier-associated cells play a crucial role in modulating the immune response at the BBB. Moreover, our recent findings suggest that interaction with an inflamed BBB drives loss-of-function and a pro-inflammatory shift in brain-infiltrating regulatory T cells (Baeten et al., 2024), potentially weakening their ability to control local antigen presentation (through diminished expression of inhibitory molecules) and activation of autoreactive T cells (via a switch from anti-inflammatory to pro-inflammatory cytokine secretion). This may promote sustained CNS antigen presentation, leading to activation of naive or low-affinity autoreactive T cells, and ultimately facilitating the diversification of the autoimmune response through epitope spreading in MS. Altogether, the compromised BBB thereby serves as a secondary, yet powerful, amplifier of immune activation, fueling a vicious cycle where increased antigen release, heightened APC activity, and continuous T cell migration and entry exacerbate CNS tissue damage (Zierfuss et al., 2024).

Finally, at the forefront of immune surveillance is the meningeal barrier, which consists of the dura mater, arachnoid mater, and pia mater (Magliozzi et al., 2023). Enveloping the entire brain and spinal cord, the meninges form a highly vascularized and lymphatically connected structure that serves as a key interface between the CNS and the immune system (Jain and Yong, 2022; Magliozzi et al., 2023). Emerging research indicates that this barrier is not only a site of immune cell infiltration but also a critical location for antigen presentation and T cell activation in MS (Kivisakk et al., 2009). Research has shown that CD4^+^ T cells accumulate within the leptomeninges before infiltrating into deeper CNS compartments (Kivisakk et al., 2009; Magliozzi et al., 2023). Within the subarachnoid space, these T cells interact with dendritic cells, meningeal macrophages, and B cells, indicating antigen recognition and activation (Kivisakk et al., 2009; Magliozzi et al., 2023). This suggests that rather than being reactivated within the brain parenchyma, autoreactive T cells undergo a crucial activation step at the meningeal barrier, which may serve as an important additional site for CNS-targeted immune responses. In fact, continuous sampling of CNS-derived antigens by APC within the meninges allows for efficient presentation to infiltrating T cells, reinforcing local immune activation (Jain and Yong, 2022; Magliozzi et al., 2023). This environment facilitates the reactivation of myelin-specific T cells and promotes sustained inflammation, contributing to MS progression and highlighting the role of the meninges as a dynamic immunological niche in MS.

**Modulation of antigen presentation by current disease-modifying therapies (DMTs):** There are several observations showing that current DMTs to manage MS can affect antigen presentation at CNS barriers. For example, fingolimod promotes BBB integrity through upregulation of the tight junction protein claudin-5 and downregulation of vascular cell adhesion molecule 1 on brain endothelial cells, thereby reducing BBB permeability and leukocyte adhesion (Jakimovski et al., 2024). Interferon-β lowers MHC-class II expression and surface co-stimulatory molecules, impairing APC-driven T cell activation. Dimethyl fumarate directly reduces B cell expression of antigen-presenting and co-stimulatory molecules while inhibiting pro-inflammatory cytokine production, thereby further impairing APC function (Jakimovski et al., 2024). Anti-CD20 monoclonal antibodies (e.g., ocrelizumab/rituximab) deplete circulating CD20⁺ B cells, which serve as potent APCs, thereby reducing T cell priming and pro-inflammatory cytokine secretion (Jakimovski et al., 2024). Finally, natalizumab prevents lymphocyte adhesion and CNS entry by blocking the α4 subunit of the very late antigen-4 receptor, which normally binds to vascular cell adhesion molecule 1 to enable migration of immune cells across the BBB (Jakimovski et al., 2024). Natalizumab also indirectly limits APC infiltration and antigen presentation within the CNS. Thus, these findings demonstrate that current DMTs exert significant effects at the CNS interfaces, particularly by altering antigen presentation and immune cell trafficking, limiting T-cell reactivation and neuroinflammation in MS.

**Conclusion:** We propose a unified, spatiotemporal model in which autoreactive T cells undergo stepwise reactivation at distinct brain barrier sites, each contributing uniquely to the immunopathology of MS. The sequence likely begins at the BCSFB, where CP epithelial cells and resident APCs facilitate early antigen presentation and immune cell recruitment. This is followed by inflammation-induced disruption of the BBB, allowing for enhanced immune cell infiltration and further antigen presentation by endothelial cells, pericytes, and infiltrating APCs. Finally, the meninges act as a critical site for sustained T cell reactivation, where ongoing antigen sampling by dendritic cells, macrophages, and B cells supports chronic inflammation and parenchymal invasion. This collectively highlights the brain barriers not as passive interfaces, but as dynamic immunological environments where diverse cell types interact to facilitate antigen presentation. Indeed, beyond dendritic cells and B cells, barrier-associated epithelial, endothelial, and perivascular cells also up-regulate MHC and costimulatory molecules during inflammatory episodes and can drive T cell activation. Dendritic cells remain key both for initial peripheral priming and for reactivation of autoreactive T cells at brain barriers, while B cells not only secrete pathogenic antibodies but also act as potent APCs for CD4^+^ T cells and secrete pro-inflammatory cytokines such as IL-6 and granulocyte-macrophage colony-stimulating factor (Jain and Yong, 2022). The significance of this dual functionality is underscored by the clinical efficacy of anti-CD20 therapies, effectively reducing relapse rates and slowing disability progression in MS (Jain and Yong, 2022). Future research should focus on strategies to modulate B-cell antigen presentation at the brain barriers, with the goal of developing therapies that fine-tune the APC activity of B cells, while preserving their regulatory functions.

While our unified, spatiotemporal model positions the choroid plexus, BBB, and meninges as sequential checkpoints for autoreactive T-cell reactivation, major questions remain: which non-professional APCs can independently sustain local T-cell activation; how does the antigen repertoire shift across barriers during epitope spreading; and in what order do different T-cell subsets traverse these interfaces. Additionally, the initial peripheral T cell priming and the specific antigens involved remain unresolved. Future investigations should focus on unraveling the relative contributions and interactions of these diverse APCs at different brain barrier sites and how DMTs reprogram APC–T-cell crosstalk. By pinpointing which APC subsets initiate and sustain autoreactive T-cell activation in MS, these focused efforts will lay the groundwork for next-generation, barrier-targeted immunotherapies and improved patient outcomes.

*This work was supported by Belgian Charcot Foundation (to BB)*.
